# High Pain Catastrophizing Scale Predicts Lower Patient-Reported Outcome Measures in the Foot and Ankle Patient

**DOI:** 10.1177/19386400221093865

**Published:** 2022-05-23

**Authors:** Andrea Veljkovic, Oliver Gagne, Monther Abuhantash, Alastair S. E. Younger, Michael Symes, Murray J. Penner, Kevin J. Wing, Khalid A. Syed, Johnny Lau

**Affiliations:** Department of Orthopedics, Footbridge Centre for Integrated Foot and Ankle Care, St. Paul’s Hospital, The University of British Columbia, Vancouver, BC, Canada; Department of Orthopedics, Footbridge Centre for Integrated Foot and Ankle Care, St. Paul’s Hospital, The University of British Columbia, Vancouver, BC, Canada; Department of Orthopedics, University of Manitoba, Winnipeg, MB, Canada; Department of Orthopedics, Footbridge Centre for Integrated Foot and Ankle Care, St. Paul’s Hospital, The University of British Columbia, Vancouver, BC, Canada; Department of Orthopedics, Footbridge Centre for Integrated Foot and Ankle Care, St. Paul’s Hospital, The University of British Columbia, Vancouver, BC, Canada; Department of Orthopedics, Footbridge Centre for Integrated Foot and Ankle Care, St. Paul’s Hospital, The University of British Columbia, Vancouver, BC, Canada; Department of Orthopedics, Footbridge Centre for Integrated Foot and Ankle Care, St. Paul’s Hospital, The University of British Columbia, Vancouver, BC, Canada; Arthritis Program, Toronto Western Hospital and Research Institute, University Health Network, Department of Surgery, University of Toronto, Toronto, ON, Canada; Arthritis Program, Toronto Western Hospital and Research Institute, University Health Network, Department of Surgery, University of Toronto, Toronto, ON, Canada

**Keywords:** catastrophization, foot, ankle, pain, function, disability

## Abstract

**Background:**

Postoperative outcomes may be affected by the patient’s preoperative morbidity. It is hypothesized that patient’s pain catastrophization prior to foot and ankle surgery may affect their patient-reported outcomes. Methods: This study prospectively assessed a consecutive cohort of 46 patients undergoing foot and ankle reconstruction to describe the relationship between Pain Catastrophizing Scale (PCS) and patient-reported outcomes measured by 12-item Short Form Health Survey and Foot and Ankle Outcome Score (FAOS).

**Results:**

The 1-year postoperative FAOS pain, activities of daily living, and quality of life scores correlated significantly with all baseline PCS subcategories. We found that the mental domain of the SF-12 had a statistically significant correlation with the rumination and helplessness PCS subcategories.

**Conclusion:**

This study showed a significant association between a high preoperative PCS and a worse 1-year FAOS. As such, catastrophization could be screened for and potentially treated preoperatively to improve patient-reported outcomes in elective foot and ankle surgery.

**Level of Evidence:**

Therapeutic, Level III Evidence

## Introduction

Catastrophization has already been identified to impact outcomes in total knee arthroplasty patients.^1,2^ No previous studies have analyzed the relationship between catastrophization and foot and ankle surgery outcomes. Currently, patient-reported outcome scores are often used as a tool to compare different treatments in foot and ankle surgery. Current outcome scores, such as the 12-item Short Form Health Survey, typically bundle physical, mental, and functional components together.^3^ There are more disease-specific outcome scores such as the Foot and Ankle Outcome Score (FAOS)^4^ and the Ankle Osteoarthritis Scale.^5^ Historically, certain tools have had greater validity than others.^6^ Newer patient-reported outcome measures use computer-based question algorithm,^7,8^ and they have been reported to be more precise and responsive to patients’ outcomes.^9^ It is not well understood what effect preoperative catastrophization may have on outcome measures in foot and ankle surgery. To our knowledge, this is the first study to look at this association.

Patients’ health care experience may be modulated by their understanding of their preoperative disability along with their overall coping strategy and life experiences.^10^ Some patients’ approach to problems focuses substantially on their disability and recovering from the trauma or the progression of their recovery. With more and more focus on patient-reported outcomes defining treatment success and access to care,^11,12^ there may be patients with apparently similar clinical and radiological postoperative results but different patient-reported outcome measures (PROMs) scores. There has also been an increasing recognition of the importance of mental health and psychological wellness as well as having a more patient-centered approach to the delivery of care.^2^

**Figure 1.**
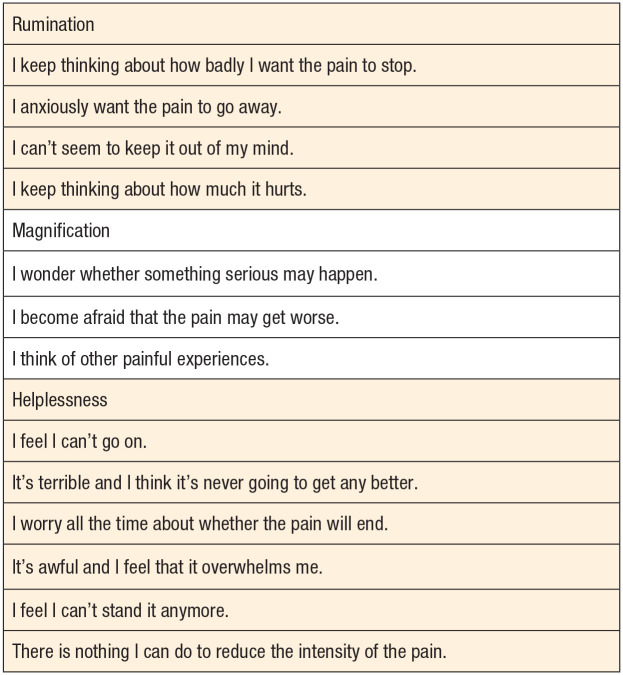
Questions of the Pain Catastrophizing Scale. A 13-item questionnaire addressing thoughts and feelings experienced by patients in pain. Reference to Sullivan et al.

A series of articles on Pain Catastrophizing Scale (PCS) have been published in the past 20 years by Sullivan et al.^13,14^ Pain Catastrophizing Scale is a 13-item questionnaire addressing thoughts and feelings experienced by patients in pain (Figure 1).^15,16^ Modifiable psychological factors (mindfulness, resilience, etc) have been previously identified that are negatively associated with patient outcomes after a total knee replacement.12 Glazebrook et al demonstrated that patients suffering from end-stage ankle arthritis had similar pain and disability as patients suffering from hip arthritis as well as a negative impact on preoperative mental health.^17^ Hence it is possible that a poor psychological state could be playing an even bigger role in foot and ankle surgery.

As such, risk factors targeted by preoperative psychological intervention could be used in high-risk patients to modulate postoperative perceived pain.^18^ A series of multiweek intervention programs have been reported to decrease catastrophizing scores up to 40%.^19,20^

### Objectives

This study analyzes the correlation between patient’s preoperative catastrophizing scale and their 1-year postoperative FAOS domain scores for patients undergoing a series of foot and ankle operative interventions. We hypothesize that patients with a higher preoperative PCS would report lower functional outcome postoperatively.

## Materials and Methods

### Study Design

This was a retrospective observational cohort study that used prospectively collected data of a foot and ankle group based in one tertiary referral center. All patients were assessed and treated operatively by 1 of 3 experienced, fellowship-trained, board-certified surgeons. All patients undergoing a foot and ankle operative procedure were recruited consecutively between March 2014 and August 2015 and agreed to participate in the prospective database. Procedures followed were in accordance with the ethical standards of the responsible committee on human experimentation, institutional and national (ethics approval number: 16-6194).

To be included in the study, patients had to be 18 years of age or older and undergo a foot and ankle surgery. This was a pragmatic study that aimed to represent a common foot and ankle clinical practice. The patients with lack of conversational English were excluded given that PCS has only been validated in English, French, and Arabic.^21^ We also excluded any patients with missing data. The treatment options were not affected by the participation in the database and were made clinically. Participants were approached by the research team and then enrolled on a voluntary basis with no compensation or incentives. Their preoperative data set was compiled along with their 1-year postoperative data set for further analysis. No power analysis was performed as this was a pilot study.

### Setting

Fifty-four patients were approached to participate in this study and 8 patients were excluded for missing the 1-year follow-up after surgery. Forty-six patients were included in the study, and their demographic information was collected including gender, age, body mass index (BMI), and side and location of their surgery (hindfoot, midfoot, or forefoot; Table 1).

**Table 1.** Demographics of the Cohort and the Subgroups.

Whole cohort			Group 1: PCS <15	Group 2: PCS ≥15	*P* value
		Value [95% CI]	Value [95% CI]	Value [95% CI]	
Gender	N	46	33	13	
	Male	16	11	5	
	Female	30	22	8	
Side					
	Left	23	18	5	
	Right	21	13	8	
	Bilateral	2	2	0	
Age (years)		54.7 [50.6-58.9]	56 [52-60]	51 [42-60]	.2
BMI (kg/m^2^)		26.2 [24.5-27.8]	26 [24-27]	28 [24-31]	.3
Location					.35
	Forefoot	12	10	2	
	Midfoot	8	5	3	
	Hindfoot	26	18	8	
Baseline PCS					
Rumination		4.00 [3.03-4.97]	2.27 (1.60-2.95)	7.77 (6.62-8.92)	
Helplessness		4.16 [2.89-5.42]	2.00 (1.33-2.67)	9.31 (7.34-11.28)	
Magnification		2.13 [1.52-2.74]	1.42 (0.90-1.95)	3.77 (2.64-4.90)	
Total		10.30 [7.71-12.88]	5.70 (4.20-7.19)	20.85 (17.62-24.07)	

Demographics of the study patients with their baseline PCS.

Abbreviations: PCS, Pain Catastrophizing Scale; CI, confidence interval; BMI, body mass index.

### Variables

Patients completed a survey administered by an independent research staff member during their preoperative visit and their 1-year postoperative visit. The survey incorporated validated outcome scores including the SF-12, the FAOS, and the PCS. The SF-12 comprised the Mental Component Summary (MCS) and the Physical Component Summary. The FAOS score can be subcategorized into other symptoms, pain, activities of daily living (ADL), sports and recreation, and quality of life (QoL). The PCS scale has 3 subcategories which are rumination, helplessness, and magnification, whose added score makes up the total PCS score. The primary outcome for this study was to assess the relationship between PCS and FAOS and its subcategories scores, since these are more condition specific. The SF-12–derived mental component and physical component are both generic health-related QoL surveys, and so are not specific for foot and ankle pathologies.

### Analysis

Univariate and multivariate analyses were conducted using the demographics (age, gender, BMI, employment status) and the PCS variables (rumination, helplessness, magnification, and total PCS score). The preoperative FAOS score was controlled as well. A univariate analysis was performed to establish which of these preoperative variables correlate with the postoperative FAOS subdomain scores. Each of the variables that had a *P* value <.25 was included into the multivariate analysis. This high *P* value was selected to include more variables with potential signal in the multivariate analysis. An alpha level of 5% for statistical significance was applied for reporting all statistical analyses. The same process was repeated for the SF-12 physical and mental scores.

In addition, we will also be looking at the minimally important differences (MIDs) in the FAOS subdomain scores. These have been previously shown to be mostly ranging from above 4 to below 16.^22^ Ranges for the pain, symptoms, ADL, QoL, and sports and recreation domains are 5.8 to 10.2, 0.3 to 6.9, 8.3 to 10.3, 7.4 to 11.1, and 7.0 to 15.7, respectively.

### Subgroup Analysis

According to the main PCS validation study, patients with PCS value of <15 are considered within the normal limit of catastrophization, whereas those with PCS score of ≥15 are considered catastrophizers.^15^ Further studies categorized patients with PCS score of ≥30 to be severe catastrophizers.^23^ As such, we have divided our patient cohort into 2 groups, low-risk (PCS <15) and high-risk (PCS ≥15) catastrophizers. In our cohort of patients, 33 belonged to the low-risk group, while 13 belonged to the high-risk group (Table 1). Both groups were balanced as far as gender, sides, age, and BMI. Further analysis comparing both groups for both the preoperative and the postoperative SF-12 and FAOS was done using Student’s *t* test.

## Results

### Participants

A cohort of 46 patients had a completed data set for both the preoperative and the 1-year follow-up visits. The cohort had a mean age of 54.72 years (95% confidence interval [CI]: 50.55-58.88), a majority of female 30/46 (65%), a minority employed at the preoperative visit 19/46 (41%), and a mean BMI (kg/m^2^) of 26.2 (95% CI: 24.5-27.8) as seen in Table 1.

For the 1-year postoperative FAOS symptoms domain (Table 2), we can see that only the PCS rumination (*P* = .11) passed the threshold of *P* value <.25 in the univariate analysis. As such it was brought into the multivariate analysis along with preoperative FAOS symptoms, but that did not show any statistical significance (*P* = .84).

**Table 2.** One-Year Postoperative FOAS Scores With Preoperative PCS.

	FAOS symptoms		FAOS pain		FAOS ADL		FAOS sport		FAOS QoL	
Univariate analysis										
	Effect [95% CI]	*P* value	Effect [95% CI]	*P* value	Effect [95% CI]	*P* value	Effect [95% CI]	*P* value	Effect [95% CI]	*P* value
Age	0.35 [−0.14-0.83]	.15	−0.04 [−0.44-0.36]	.85	−0.05 [−0.37-0.27]	.75	−0.08 [−0.67-0.5]	.78	0.43 [−0.13-0.98]	.13
Gender (female vs male)	4.52 [−10.15-19.2]	.54	−4.43 [16.24-7.37]	.45	0.95 [−8.73-10.63]	.84	1.1 [−16.35-18.56]	.9	−1.33 [−18.36-15.7]	.88
Currently employed	−7.08 [−21.52-7.37]	.33	2.21 [−8.93-13.36]	.69	4.55 [−4.41-13.5]	.31	5.65 [−10.8-22.1]	.49	−3.82 [−19.96-12.32]	.64
BMI (kg/m^2^)	−0.8 [−2.17-0.57]	.25	−0.87 [−1.91-0.17]	.1	−0.56 [−1.41-0.3]	.2	−0.18 [−1.77-1.4]	.82	0.03 [−1.49-1.55]	.97
PCS rumination	−1.84 [−4.09-0.4]	.11	−2 [−3.78 to −0.23]	.03	−1.85 [−3.27 to −0.43]	.01	−1.6 [−4.31-1.11]	.24	−2.55 [−5.12-0.02]	.05
PCS helplessness	−0.95 [−2.65-0.75]	.27	−1.5 [−2.81 to −0.19]	.03	−1.81 [−2.8 to −0.81]	.001	−1.15 [−3.17-0.86]	.25	−1.51 [−3.45-0.42]	.12
PCS magnification	0.32 [−3.26-3.89]	.86	0.49 [−2.38-3.37]	.73	−0.6 [−2.94-1.75]	.61	1.37 [−2.85-5.58]	.52	1.41 [−2.7-5.51]	.49
PCS total	−0.48 [−1.33-0.38]	.27	−0.63 [−1.31-0.05]	.07	−0.75 [−1.27 to −0.22]	.01	−0.44 [−1.46-0.59]	.4	−0.66 [−1.64-0.33]	.19
Multivariate analysis										
Baseline PCS rumination	−0.24 [−2.64-2.15]	.84	−7.6 [−12.21 to −2.98]	.002	−4.67 [−8.53 to −0.82]	.02	−1.53 [−4.96-1.91]	.37	−11.59 [−17.57 to −5.61]	.0004
Baseline PCS helplessness			−6.73 [−11.62 to −1.83]	.01	−5.89 [−9.94 to −1.84]	.01	1.17 [−1.63-3.96]	.4	−9.65 [−15.42 to −3.89]	.002
Baseline PCS total			5.44 [1.89-9]	.004	3.74 [0.78-6.7]	.02			8.54 [4.16-12.92]	.0003

One-year postoperative FAOS scores with univariate and multivariate analysis using the preoperative Pain Catastrophizing Scale.

Abbreviations: FAOS, Foot and Ankle Outcome Score; PCS, Pain Catastrophizing Scale; ADL, activities of daily living; CI, confidence interval; BMI, body mass index; QoL, quality of life.

Similar univariate and multivariate analyses were done for the 1-year postoperative FAOS pain, ADL, sport, and QoL domains (Table 2). The FAOS pain, ADL, and QoL domains showed a statistically significant association with rumination, helplessness, and PCS total domains in the multivariate analysis. On the other hand, the FAOS sport domain showed no association with any of the PCS domains.

The secondary outcome analyzed in this study (SF-12) is less disease specific. For the mental component 1 year postoperative, the univariate analysis showed statistically significant correlation with rumination (*P* = .01), helplessness (*P* = .001), and PCS total (*P* = .01). These 3 variables were included in the multivariate analysis along with preoperative SF-12 mental component. The multivariate analysis showed no statistically significant correlation with the variables of interest: rumination (*P* = .87), helplessness (*P* = .07), and PCS total (*P* = .31). This can be seen in Table 3.

**Table 3.** One-Year Postoperative SF-12 With Preoperative PCS.

	SF-12 mental		SF-12 physical	
Univariate analysis				
	Effect [95% CI]	*P*-value	Effect [95% CI]	*P*-value
Age	0.15 [−0.04-0.34]	.11	−0.21 [−0.44-0.03]	.08
Gender (female vs male)	−3.14 [−8.69-2.4]	.26	−1.96 [−8.88-4.97]	.57
Currently employed	1.83 [−3.55-7.21]	.5	4.26 [−2.08-10.6]	.18
BMI (kg/m^2^)	−0.23 [−0.71-0.25]	.34	−0.53 [−1.13-0.06]	.08
PCS rumination	−1.03 [−1.84 to −0.21]	.01	−0.79 [−1.85-0.26]	.13
PCS helplessness	−1.05 [−1.61 to −0.48]	.001	−0.7 [−1.47-0.07]	.07
PCS magnification	−0.54 [−1.87-0.79]	.42	0.17 [−1.48-1.83]	.83
PCS total	−0.44 [−0.74 to −0.14]	.01	−0.28 [−0.67-0.12]	.16
Multivariate analysis				
Baseline PCS rumination	−0.13 [−1.79-1.53]	.87	−2.28 [−4.81-0.26]	.08
Baseline PCS helplessness	−1.49 [−3.12-0.14]	.07	−2.1 [−4.73-0.52]	.11
Baseline PCS total	0.6 [−0.6-1.81]	.31	1.53 [−0.38-3.45]	.11

One-year postoperative SF-12 scores with univariate and multivariate analysis using the preoperative Pain Catastrophizing Scale.

Abbreviations: 12-item Short Form Health Survey; PCS, Pain Catastrophizing Scale; CI, confidence interval; BMI, body mass index.

For the physical component 1 year postoperative, the univariate analysis showed statistically no significant correlation with the variables of interest. Since rumination (*P* = .13), helplessness (*P* = .07), and PCS total (*P* = .16) were below the threshold (*P* < .25), these 3 variables were included in the multivariate analysis along with preoperative SF-12 physical component. The multivariate analysis showed no statistically significant correlation with the variables of interest: rumination (*P* = .08), helplessness (*P* = .11), and PCS total (*P* = .11; Table 3).

The PCS subscores are different (*P* < .05) between the 2 groups (Table 4). There are no statistically significant differences between the preoperative baseline FAOS and SF-12 components. Postoperatively, there are statistically significant differences between the 2 groups in the SF-12 mental component (7 points, *P* = .009), FAOS pain domain (5 points, *P* = .01), ADL (9 points, *P* = .003).

**Table 4.** Baseline and 1-Year Scores Between Low-Risk and High-Risk Catastrophizers.

			Group 1: PCS <15	Group 2: PCS ≥15	*P*-value
			Value [95% CI]	Value [95% CI]	
Baseline	PCS	Rumination	2.27 [1.60-2.95]	7.77 [6.62-8.92]	>.005
		Helplessness	2.00 [1.33-2.67]	9.31 [7.34-11.28]	>.005
		Magnification	1.42 [0.90-1.95]	3.77 [2.64-4.90]	.73
		Total	5.70 [4.20-7.19]	20.85 [17.62-24.07]	>.005
Baseline	SF-12	Mental	54.76 [51.77-57.76]	48.67 [42.56-54.79]	.08
		Physical	37.65 [33.83-41.47]	34.15 [28.19-40.10]	.4
	FAOS	Other symptoms	10.18 [8.19-12.17]	13.77 [10.40-17.14]	.08
		Pain	13.00 [10.93-15.07]	15.54 [10.42-20.66]	.3
		ADL	20.36 [16.38-24.35]	23.77 [14.62-32.92]	.5
		Sports	11.97 [9.89-14.05]	11.54 [7.04-16.03]	.9
		QoL	11.00 [9.86-12.14]	11.92 [10.16-13.69]	.4
One-year postoperative	SF-12	Mental	55.36 [52.90-57.82]	47.70 [42.05-53.35]	.009
		Physical	44.56 [40.97-48.15]	39.75 [33.45-46.06]	.2
	FAOS	Other symptoms	7.42 [5.37-9.48]	10.08 [5.95-14.20]	.2
		Pain	6.42 [4.78-8.06]	11.92 [6.88-16.96]	.01
		ADL	7.09 [4.75-9.43]	17.00 [9.22-24.78]	.003
		Sports	8.18 [6.49-9.87]	9.46 [5.72-13.20]	.5
		QoL	6.85 [5.63-8.07]	8.54 [5.42-11.65]	.2

Comparison of patient-reported baseline scores (PCS, FAOS, and SF-12) and 1-year postoperative scores (FAOS and SF-12) between 2 groups of patients based on their PCS scores: low-risk catastrophizers (PCS <15) and high-risk catastrophizers (PCS >15).

Abbreviations: PCS, Pain Catastrophizing Scale; CI, confidence interval; 12-item Short Form Health Survey; FAOS, Foot and Ankle Outcome Score; ADL, activities of daily living; QoL, quality of life.

## Discussion

This study demonstrates that an increase in the preoperative PCS score correlates to a worse result as reflected by the lower FAOSs. Looking at the correlation with FAOS, the pain, ADL, and QoL domains showed strong positive correlation with rumination, helplessness, and total PCS score. This is the first foot and ankle study to report a correlation of this kind. Although this is a small study, the results suggest it may be important to administer a preoperative PCS to stratify patients’ expected postoperative PROMs. This has the potential of changing the operative selection in foot and ankle surgery, as high-risk catastrophizing patients would be stratified and referred to psychologist for intervention and reassessed later when the scores trend lower, hence becoming better operative candidates leading them to have better postoperative PROMs.

When addressing specifically the SF-12, it was noted that the multivariate analysis for both the mental and the physical scores did not show statistically significant correlation with catastrophizing. This is not surprising as general health scores maybe less responsive than anatomic-specific instruments with respect to foot and ankle operative intervention.

When looking at the stratification piece with the 2 groups, it is remarkable to notice that the catastrophizers of this cohort with PCS ≥15 had the same preoperative SF-12 and FAOS, but had statistically significant different scores 1 year postoperatively. There are statistically significant differences between the 2 groups in the SF-12 mental component (7.6 points, *P* = .009), FAOS pain domain (5.5 points, *P* = .01), and ADL (9.9 points, *P* = .003). Although these are statistically significantly different, only ADL reaches the MID of 8.3 to 10.3 based on a recent publication of the MID of the FAOS domains for hallux valgus surgery.^22^ The pain domain difference (5.5 points) did not meet the MID ranging from 5.8 to 10.2.

Overall, our results correlate with a previous study from Dunn et al that identified catastrophization, anxiety, and depression as important players in modulating postoperative pain during the inpatient period.^18^ Our results point in the same direction as Yakobov et al who noted that pain catastrophization correlated significantly to the postoperative health-related quality of life (HRQoL) over other variables (demographic variables, comorbid health conditions, baseline HRQoL, and postoperative reductions in pain, joint stiffness, and physical disability).^24^ A similar trend was observed by Wright et al supporting that catastrophizers (PCS >30) would be more likely to have a longer length of hospital stay after total joint surgery.^25^ This study had only 1 patient with PCS scores >30 so this may underestimate the correlation with SF-12 and FAOS. It may be that the threshold where a patient is considered catastrophizer is also disease specific.

Interestingly, a study by Goh et al noted that in patients undergoing hallux valgus surgery a low SF-36 MCS score correlated with poorer short-term outcomes at 6 months compared with non-distressed patients.^26^ However, these differences resolved at 2 years, which may imply that preoperative psychological distress may not have a significant impact on long-term postoperative outcomes.

The role or potential benefit of preoperative psychological referral and intervention in catastrophizers has not been extensively studied. It is an experimental suggestion that could be further explored in a future study. In addition, pain-coping skills training has been proposed to be beneficial in conjunction with medical treatment in managing pain in patients with arthritis.^27^

There are many ways to interpret the results and stimulate further discussion. This is a small study showing association and not a causality. It is not clear whether patients with high catastrophizing scores rate worse PROMs regardless of their true disability. Do patients with high PCS scores request operative treatment for lesser pathology? This study found that the high-risk subgroup had worse postoperative functional scores compared with the low-risk subgroup in spite of similar preoperative scores. As such, it would be prudent to consider incorporating this in preoperative patient selection and stratification with the aim of ensuring that patients receive the interventions they need to alleviate the factors causing them to catastrophize, potentially resulting in them having favorable postoperative PROMs.

### Bias

As an attempt to minimize the selection bias, this study was designed as a consecutive enrollment until the goal of *N* = 40 was reached. There was no power analysis. The heterogeneity of the location of the surgery (see Table 1) was a concern.

However, this is representative of the typical diversity within a community foot and ankle practice. Although stratification of the study cohort into hindfoot, midfoot, and forefoot procedures may have been helpful, it could not be performed due to the small size of the stratified groups.

## Conclusion

This study strongly suggests that postoperative patient-reported outcomes (SF-12, FAOS) in foot and ankle surgery patients correlate with preoperative pain catastrophization. This may change patient selection or highlight the utility of preoperative counseling administered before surgery to patients with foot and ankle pathology. These findings highlight the potential need for certain outcome measures to be adjusted or updated to incorporate measures of catastrophization. Further large-scale studies with appropriate power and stratification by surgery to better define the impact of catastrophization in foot and ankle surgery patients are warranted.

## References

[1] Bierke S Petersen W . Influence of anxiety and pain catastrophizing on the course of pain within the first year after uncomplicated total knee replacement: a prospective study. Arch Orthop Trauma Surg. 2017(12):1735-1742. doi:10.1007/s00402-017-2797-5. 28965133

[2] Ellis HB Howard KJ Khaleel MA Bucholz R . Effect of psychopathology on patient-perceived outcomes of total knee arthroplasty within an indigent population. J Bone Joint Surg Am. 2012;94(12):E84. doi:10.2106/JBJS.K.00888 22717836

[3] Ware J Jr. Kosinski M Keller SD . A 12-Item Short-Form Health Survey: construction of scales and preliminary tests of reliability and validity. Med Care. 1996;34(3):220-233. 8628042 10.1097/00005650-199603000-00003

[4] Roos EM Brandsson S Karlsson J . Validation of the foot and ankle outcome score for ankle ligament reconstruction. Foot Ankle Int. 2001;22(10):788-794. doi:10.1177/107110070102201004. 11642530

[5] Domsic RT Saltzman CL . Ankle osteoarthritis scale. Foot Ankle Int. 1998;19(7):466-471. doi:10.1177/107110079801900708. 9694125

[6] Madeley NJ Wing KJ Topliss C Penner MJ Glazebrook MA Younger AS . Responsiveness and validity of the SF-36, Ankle Osteoarthritis Scale, AOFAS Ankle Hindfoot Score, and Foot Function Index in end stage ankle arthritis. Foot Ankle Int. 2012;33(1):57-63. doi:10.3113/fai.2012.0057. 22381237

[7] Ho B Houck JR Flemister AS , et al. Preoperative PROMIS scores predict postoperative success in foot and ankle patients. Foot Ankle Int. 2016;37(9):911-918. doi:10.1177/1071100716665113. 27530986

[8] Hung M Baumhauer JF Latt LD Saltzman CL SooHoo NF Hunt KJ . Validation of PROMIS (R) Physical Function computerized adaptive tests for orthopaedic foot and ankle outcome research. Clin Orthop Relat Res. 2013;471(11):3466-3474. doi:10.1007/s11999-013-3097-1. 23749433 PMC3792246

[9] Hung M Saltzman CL Voss MW , et al. Responsiveness of the PROMIS, NDI and ODI Instruments in Patients with Spinal Disorders. Spine J. 2019;34-40. doi:10.1016/j.spinee.2018.06.355. 29969730 PMC6309663

[10] Li CC Shun SC . Understanding self care coping styles in patients with chronic heart failure: a systematic review. Eur J Cardiovasc Nurs. 2016;15(1):12-19. doi:10.1177/1474515115572046. 25681369

[11] Callahan LF . The history of patient-reported outcomes in rheumatology. Rheum Dis Clin North Am. 2016;42(2):205-217. doi:10.1016/j.rdc.2016.01.012. 27133485

[12] Wright AA Hensley CP Gilbertson J Leland JM III Jackson S . Defining patient acceptable symptom state thresholds for commonly used patient reported outcomes measures in general orthopedic practice. Manual Therapy. 2015;20(6):814-819. doi:10.1016/j.math.2015.03.011. 25843266

[13] Pavlin DJ Sullivan MJ Freund PR Roesen K . Catastrophizing: a risk factor for postsurgical pain. Clin J Pain. 2005;21(1):83-90. 15599135 10.1097/00002508-200501000-00010

[14] Wideman TH Sullivan MJ . Reducing catastrophic thinking associated with pain. Pain Management. 2011;1(3):249-256. doi:10.2217/pmt.11.14. 24646391

[15] Sullivan MJL Bishop SR Pivik J . The Pain Catastrophizing Scale: development and validation. Psychol Assess. 1995;7:524-532. doi:10.1037/1040-3590.7.4.524.

[16] Keefe FJ Abernethy APLCC . Psychological approaches to understanding and treating disease-related pain. Annu Rev Psychol. 2005;56:601-630. doi:10.1146/annurev.psych.56.091103.070302. 15709948

[17] Glazebrook M Daniels T Younger A , et al. Comparison of health-related quality of life between patients with end-stage ankle and hip arthrosis. J Bone Joint Surg. 2008;90(3):499-505. doi:10.2106/jbjs.f.01299. 18310699

[18] Dunn LK Durieux ME Fernandez LG , et al. Influence of catastrophizing, anxiety, and depression on in-hospital opioid consumption, pain, and quality of recovery after adult spine surgery. J Neurosurg Spine. 2018;28(1):119-126. doi:10.3171/2017.5.spine1734. 29125426 PMC5772650

[19] Jensen MP Turner JA Romano JM . Changes in beliefs, catastrophizing, and coping are associated with improvement in multidisciplinary pain treatment. J Consult Clin Psychol. 2001;69(4):655-662. 11550731 10.1037//0022-006x.69.4.655

[20] Sullivan MJ Stanish W Sullivan ME Tripp D . Differential predictors of pain and disability in patients with whiplash injuries. Pain Res Manag. 2002;7(2):68-74. 12185370 10.1155/2002/176378

[21] Terkawi AS Sullivan M Abolkhair A , et al. Development and validation of Arabic version of the pain catastrophizing scale. Saudi J Anaesth. 2017;11(suppl 1):S63-S70. doi:10.4103/sja.SJA_130_17. 28616005 PMC5463568

[22] Desai S Peterson AC Wing K , et al. Minimally important difference in the foot and ankle outcome score among patients undergoing hallux valgus surgery. Foot Ankle Int. 2019. doi:10.1177/1071100719831392 30873859

[23] Sullivan MJ Stanish W Waite H Sullivan M Tripp DA . Catastrophizing, pain, and disability in patients with soft-tissue injuries. Pain. 1998;77(3):253-260. 9808350 10.1016/S0304-3959(98)00097-9

[24] Yakobov E Stanish W Tanzer M Dunbar M Richardson G Sullivan MJL . The prognostic value of pain catastrophizing in health-related quality of life judgments after Total knee arthroplasty. Health Qual Life Outcome. 2018;16(1):126. doi:10.1186/s12955-018-0955-2. PMC6006578 29914521

[25] Wright D Hoang M Sofine A Silva JP Schwarzkopf R . Pain catastrophizing as a predictor for postoperative pain and opiate consumption in total joint arthroplasty patients. Arch Orthop Trauma Surg. 2017;137(12):1623-1629. doi:10.1007/s00402-017-2812-x. 28975493

[26] Goh GS Thever Y Tay AYW Rikhraj IS Koo K . Can patients with psychological distress achieve comparable functional outcomes and satisfaction after hallux valgus surgery? a 2-year follow-up study. Foot Ankle Surg. 2020;27:660-664 . doi:10.1016/j.fas.2020.08.011. 32917525

[27] Broderick JE Keefe FJ Bruckenthal P , et al. Nurse practitioners can effectively deliver pain coping skills training to osteoarthritis patients with chronic pain: a randomized, controlled trial. Pain. 2014;155(9):1743-1754. doi:10.1016/j.pain.2014.05.024. 24865795 PMC4171086

